# Homoeriodictyol, targeting the bitter taste receptor TAS2R14, lowers the secretion of pro-inflammatory chemokines upon treatment with SARS-CoV-2 peptide pools in human peripheral blood mononuclear cells

**DOI:** 10.3389/fimmu.2026.1771794

**Published:** 2026-02-03

**Authors:** Barbara Danzer, Gaby Andersen, Kristin Kahlenberg, Veronika Somoza

**Affiliations:** 1 School of Life Sciences, Technical University of Munich, Freising, Germany; 2 Leibniz Institute for Food Systems Biology, Technical University of Munich, Freising, Germany; 3 Institute of Physiological Chemistry, Faculty of Chemistry, University of Vienna, Vienna, Austria

**Keywords:** bitter, CCL2 (MCP-1), CCL7 (MCP-3), COVID-19, CXCL9 (MIG), cytokine, HPBMC (human peripheral blood mononuclear cells), immune

## Abstract

Excessive cytokine production is a major complication in severe COVID-19. Treatment with antiviral drugs often elicits a bitter taste through activation of bitter taste receptors (TAS2Rs). Since ectopically expressed TAS2Rs can alter cytokine secretion, we hypothesized that homoeriodictyol (HED), a broad TAS2R ligand, modulates the cytokine response to SARS-CoV-2 peptide pools (PP) in human peripheral blood mononuclear cells (PBMCs). Treatment of PBMCs isolated from healthy donors with PP for 24 h induced the mRNA expression of CXCL9, CCL7, and CCL2, the most of 116 cytokines tested. Protein release of these chemokines was quantified by ELISA after PP treatment for 3, 6, 12, 24, and 48 h. The results identified 24 h as the optimal incubation time to distinguish PP-induced chemokine release among PBMCs, T–cells, and T–cell–depleted PBMCs. PBMCs demonstrated the highest mean fold changes of CXCL9, CCL7, and CCL2 with 12, 52, and 96, respectively. Involvement of TAS2Rs was verified (i) by co-incubation of the PBMCs with PP and HED, which decreased (*p*<0.01) the PP-induced secretion of CXCL9, CCL7, and CCL2 by a mean of 80%, 96%, and 95%, respectively, and (ii) via an siRNA knock-down approach targeting *TAS2R14*. *TAS2R14* knock-down increased (*p*<0.05) the CXCL9, CCL7, and CCL2 release after 24 h of PP incubation by 33%, 34%, and 29%, respectively. These results reveal TAS2Rs on human PBMCs being functionally active in the chemokine immune response to SARS-CoV-2-specific peptides, with the broadly tuned TAS2R14 as a promising target for anti-inflammatory immune system regulation in viral infections.

## 1 Introduction

Severe acute respiratory syndrome coronavirus 2 (SARS−CoV-2), the etiological agent of coronavirus disease of 2019 (COVID-19), frequently triggers a dysregulated host immune response in severe SARS-CoV-2 patients (1, 2), which can culminate in a hyperinflammatory cytokine storm (3). This phenomenon is characterized by elevated circulating levels of pro−inflammatory cytokines, including interleukin−6 (IL−6), tumor necrosis factor−α (TNF−α), and interferon−γ (IFN−γ), as well as chemokines such as C-C motif chemokine ligand 2 (CCL2, monocyte chemotactic protein-1, MCP-1), C-C motif chemokine ligand 7 (CCL7, monocyte chemotactic protein-3, MCP-3), and C-X-C motif chemokine ligand 9 (CXCL9, monokine induced by gamma-interferon, MIG) (4–7). Chemokines are a subgroup of cytokines that contribute to the cytokine storm by recruiting immune cells. Among these, the three chemokines CXCL9, CCL7, and CCL2 have been repeatedly associated with COVID-19 severity and mortality (5–7). Importantly, CCL2 has been recognized as a central mediator of immune cell recruitment and tissue infiltration in severe COVID-19, contributing to immunopathology and lung injury (8, 9). Elevated levels of CCL7, already present in the early stages of severe disease, were found to correlate with a fatal outcome (10, 11). In addition to the increase in critically ill COVID-19 patients, elevated CXCL9 levels correlated with increased virus-specific CD4^+^T-cells (12) and depression severity in convalescent SARS-CoV-2 patients (13). CXCL9 is expressed by various immune-competent cells, including macrophages, dendritic cells, B-cells, and T-cells (14), and contributes to the infiltration of T-cells and B-cells (14). CCL2 and CCL7 are expressed by human lymphocytes and monocytes (15–17). In response to these chemokines, chemo-attraction is reported for monocytes (18), T-cells (19), NK-cells (20), and dendritic cells (21, 22). Peripheral blood mononuclear cells (PBMCs), which comprise key innate and adaptive immune populations, including approximately 61% T cells, 13% B cells, 15% NK cells, 9% monocytes, and 1% dendritic cells (23), serve as major producers and targets of cytokines systemically (24).

Concomitantly, alterations in taste function are among the most frequently reported neurological and sensory symptoms of COVID−19. A meta-analysis estimated that approximately 49% of patients with confirmed SARS-CoV-2 infection experience gustatory dysfunction during the course of illness (25). These disturbances include quantitative deficits, such as ageusia (complete loss of taste) and hypogeusia (reduced taste sensitivity), as well as qualitative changes, including dysgeusia (distorted taste), parageusia, and phantogeusia (26). Notably, bitter and metallic taste distortions have been repeatedly reported (27, 28), indicating complex disruptions in the gustatory system, including alterations in taste-receptor signaling or inflammatory modulation of gustatory pathways.

Bitter taste receptors (TAS2Rs) located in non-gustatory tissues are increasingly recognized as chemosensory regulators of immune function (29, 30). These G protein–coupled receptors are expressed in multiple non-gustatory immune and barrier cell types (30, 31), including human lung macrophages, where their activation by bitter-tasting ligands, such as strychnine and chloroquine, suppresses pro-inflammatory mediator release such as TNF-α, CCL3, and CXCL8 in response to microbial lipopolysaccharide (LPS) (32). In parallel, several antiviral drugs used clinically are known to elicit a pronounced bitter taste, a sensory property attributed to their activation of specific TAS2Rs. Oseltamivir phosphate, for example, is predicted by in silico modeling to interact with TAS2R38 (33). Tenofovir alafenamide has been shown to activate TAS2R1 and TAS2R39 (34). Pharmacological inhibition of TAS2R39 with 6-methylflavone reduced bitterness perception in 8 of 16 human subjects (34). Paxlovid, widely used for COVID-19 treatment, also induces a strong and persistent bitter taste (35), consistent with reported activation of TAS2R1 by nirmatrelvir (35) and activation of TAS2R1, TAS2R8, TAS2R13, and TAS2R14 by ritonavir (36), which are the two active pharmaceutical ingredients of paxlovid. Recent structural modeling, mutagenesis, and calcium-mobilization analyses identified two potential ritonavir-binding sites on TAS2R14 (37), underscoring the pharmacological relevance of TAS2Rs as off-target receptors for antiviral compounds.

However, the observation that some antiviral drugs have a bitter taste does not imply that the sensory quality of bitterness inherently signals antiviral activity. Individual bitter compounds may act as agonists at one or several of the 26 human TAS2Rs while simultaneously antagonizing others, resulting in complex and receptor-specific ligand interaction patterns that cannot be distinguished by the human tongue but may differentially shape immune responses at the cellular level (38–43). The bitter-masking flavanone homoeriodictyol (HED), for example, reduces the bitterness perceived from caffeine by approximately 40% (44). It is known to act as an antagonist to TAS2R31, -43, -50, and -20 (45), while simultaneously activating TAS2R14 and -39 (45, 46). On a cellular level, HED has been shown to counteract trans-resveratrol–induced suppression of IL-6 release in LPS-treated gingival fibroblasts via a TAS2R50-dependent mechanism, illustrating the capacity of TAS2R ligands to influence cytokine dynamics. Although direct evidence for HED-mediated modulation of CCL2, CCL7, or CXCL9 is currently lacking, TAS2R activation in other immune contexts affects cytokine output, supporting the rationale that HED - or chemically related TAS2R modulators - may attenuate excessive chemokine responses relevant to hyperinflammation in SARS-CoV-2 infection.

SARS-CoV-2–specific T-cell responses in PBMCs predominantly recognize epitopes derived from the spike (S), membrane (M), and nucleocapsid (N) proteins (47), and 15-mer peptide pools overlapping by 11 amino acids have been widely used to stimulate immune cells (48, 49), including in the present study. Although SARS-CoV-2 proteins have been reported to interact with TAS2R7 and TAS2R41 in large-scale viral–host interaction screens in a heterologous yeast system (50), the contribution of TAS2Rs to the inflammatory functions of primary human leukocytes during antiviral immune responses remains largely unexplored.

Given the convergence of (i) excessive cytokine production and taste loss as hallmarks of COVID-19, (ii) bitter-tasting antiviral therapeutics, (iii) TAS2Rs targeted by SARS-CoV-2 proteins, and (iv) the immunomodulatory functions of TAS2R ligands, a potential mechanistic link emerges. We therefore hypothesized that TAS2Rs represent a previously unrecognized therapeutic target for ligands capable of modulating the cytokine response to SARS-CoV-2 peptide pools (PP) in human PBMCs. To test this hypothesis, we first identified specific cytokines induced by PP in PBMCs. We then confirmed the anti-inflammatory role of TAS2R14 using reverse transcription and real-time quantitative polymerase chain reaction (RT-qPCR) and Enzyme Linked Immunosorbent Assay (ELISA), employing the TAS2R interaction partner HED and a small interfering RNA (siRNA)-mediated TAS2R14 knock-down. Understanding TAS2R-mediated immune signaling may provide novel insights into host–virus interactions and identify alternative strategies to attenuate hyperinflammation in COVID-19.

## 2 Materials and methods

### 2.1 Cell isolation

DONAS GmbH (Munich, Germany) provided buffy coats from anonymous healthy donors. PBMC isolation via density gradient centrifugation was performed according to the protocol by Miltenyi Biotech, using Leucosep Tubes with a porous barrier (Greiner Bio-One). Ficoll-Paque PLUS (Cytiva) was added to the Leucosep tubes and centrifuged at 1000 x g for 30 s. Phosphate-buffered saline (PBS) containing EDTA, pH 7.2 (autoMACS Rinsing Solution, Miltenyi Biotec) was used to dilute the buffy coat 1:4. The Ficoll-Paque PLUS was overlaid with the diluted buffy coat and centrifuged at 1000 x g for 10 min without break. The plasma layer was discarded, and the PBMC layer was collected in a new 50 mL tube. The cells were washed with autoMACS Rinsing Solution (Miltenyi Biotec) once with centrifugation at 300 x g for 10 min at RT and then twice at 200 x g for 10 min to remove platelets. The cells were united and suspended in 15 mL Gibco Roswell Park Memorial Institute 1640 Medium (RPMI 1640, Thermo Scientific) containing 5% human serum (PAN-Biotech) for erythrocyte depletion. MACSxpress Erythrocyte Depletion (Miltenyi Biotec) was performed according to the manufacturer’s protocol. To isolate untouched T-cells, the Pan T Cell Isolation Kit (Miltenyi Biotec) was used according to the manufacturer’s protocol. The depletion of T-cells was carried out utilizing human CD3^+^ Micro Beads (Miltenyi Biotec) per the manufacturer’s protocol. After isolation, the cells were counted, centrifuged at 300 x g for 10 min, and diluted to the necessary concentration. Cells were seeded at a density of 1 x 10^7^ cells/mL media.

### 2.2 Cell incubation, viability, and collection

Incubation experiments were performed with final concentrations of 0.6 µM of each PepTivator Peptide Pool (M, N, S, Miltenyi Biotec), 300 µM HED (Symrise) diluted in dimethyl sulfoxide (DMSO), and 0.1% DMSO when needed as a control. Incubation conditions were 37°C and 5% CO_2_. To evaluate the toxicity of the treatment, viability was determined using propidium iodide staining (Miltenyi Biotec) of at least 10^5^ PBMCs diluted with 200 µL PBS and analyzed with the MACSQuant Analyzer 16 (Miltenyi Biotec). Supernatant and cells were separated by centrifugation at 300 x g for 10 min. The supernatant was collected separately and frozen at -80°C.

### 2.3 RNA extraction of PBMCs

1 mL QIAzol (Qiagen) was added per 1 x 10^7^ cells. Using a 0.6 mm needle and syringe, the cells were thoroughly lysed and frozen at -80°C. After vortexing, the thawed homogenate was distributed in Eppendorf tubes, 1 mL each, and incubated for 5 min at room temperature (RT). 200 µL of chloroform was added to each tube. The tubes were vortexed for at least 15 s, then incubated for 2-3 min, and centrifuged at 12000 x g for 15 min at 4°C. The upper phase was carefully transferred into a new tube. 500 µl ice-cold isopropanol was added. The samples were thoroughly vortexed and frozen at -20°C for at least 10 min. The RNA was pelleted by centrifuging the samples at 12000 x g for 10 min at 4°C. The supernatant was discarded carefully. The pellet was washed with 1 mL 75% ethanol, turned upside down a few times, and centrifuged at 7500 x g for 5 min at 4°C. After removal of the supernatant, the pellet was air-dried. If necessary, the RNA of the same treatment sample was combined. DNA was digested using the RNase-Free DNase Set (Qiagen). According to the manufacturer’s protocol, RDD buffer and DNase I were added to the diluted RNA and shaken at 300 rpm for 1 h at 37°C. The reaction was stopped by adding 0.25 M EDTA, pH 8, and incubation at 65°C and 300 rpm for 15 min. After cooling, the RNA concentration was measured using a NanoDrop Spectrophotometer (Thermo Fisher Scientific). If necessary, the samples were stored at -80°C.

### 2.4 Reverse transcription and real-time quantitative polymerase chain reaction (RT-qPCR)

The RNA was transcribed to cDNA using the iScript gDNA Clear cDNA Synthesis Kit (Bio-Rad). The frozen RNA was thawed on ice, and all reactions were assembled on ice according to the manufacturer’s instructions. Custom RT-qPCR plates (Bio-Rad) with lyophilized housekeepers (B2M, GUSB, TBP), controls (RQ1, RQ2, PCR, gDNA, RT), and TAS2R primer pairs (published by Liszt et al., 2017 (45)) were used. Per well, 50 ng cDNA were added to SsoAdvanced Universal SYBR Green Supermix (Bio-Rad) and nuclease-free water for RT-qPCR according to the manufacturer’s instructions. The conditions used for the CFX 96 Real-Time System (Bio-Rad) were 95°C for 1 min, followed by 45 cycles of 95°C for 15 s, and 60°C for 1 min. The results were analyzed according to the ΔΔCt-method (51) (Pfaffl 2001) using Microsoft Excel.

### 2.5 Enzyme-Linked Immunosorbent Assay (ELISA)

Each chemokine of interest was quantified in the supernatant of the incubated PBMCs using its specific DuoSet ELISA Development System (R&D Systems) according to the manufacturer’s instructions. The optical density of the plate was measured immediately using a plate reader (Tecan) at 450 nm and 540 nm. The values at 540 nm were subtracted from the readings at 450 nm to correct for the plate’s optical imperfections. The average of the blank values was subtracted from all values. Using Graph Pad Prism version 10.2.3 for Windows (GraphPad Software, Boston, Massachusetts, USA), a four-parameter logistic (4-PL) curve-fit was performed with the average of the standard values. The concentration of cytokines in each well was calculated using the resulting variables of the curve-fit model. If necessary, the diluted samples were multiplied by their dilution factor before the duplicates of each sample were averaged. Not detectable values or values below the lowest level of detection were set to the lowest level of detection according to the manufacturer.

### 2.6 Knock-down of TAS2R14 by nucleofection

2 x 10^7^ PBMCs were nucleofected with 100 nM siRNA (negative control, GAPDH positive control, and s27145, Thermo Fisher Scientific). The primary cell kit P3 (Lonza) and the 4D-Nucleofector X Unit (Lonza) set to the EO-115 program were used according to the Amaxa 4D-Nucleofector Protocol for Unstimulated Human T Cells (Lonza). The PBMCs were incubated overnight at 37°C and 5% CO_2_. Due to the necessary dilution of transfection reagent recommended by the manufacturer, the final concentration of PBMCs was 5 x 10^6^ cells/mL media. The day after transfection, PBMCs were treated with and without 0.6 µM of each PepTivator Peptide Pool (M, N, S, Miltenyi Biotec) for 24 h. Viability after knock-down and 24 h treatment was tested using propidium iodine staining (Miltenyi Biotec) with the MACSQuant Analyzer 16 (Miltenyi Biotec) as described above. Using RT-qPCR as described above, the efficiency of TAS2R14 knock-down was determined. Compared to mock transfected cells, the GAPDH positive control efficiency reached 71.4% ± 4.4%, while the TAS2R14 knock-down efficiency reached 42.2% ± 4.5% (n = 11).

### 2.7 Statistics

Data are reported as mean ± standard error of the mean (SEM). Graphs were created in GraphPad Prism version 10 for Windows (GraphPad Software, Boston, Massachusetts, USA). The Shapiro-Wilk test was used to determine normality of the distribution. Paired analysis was performed, as the different treatments were evaluated per donor. Statistical significance was tested using 2-tailed, paired t-test, Wilcoxon matched-pairs signed rank test, or non-parametric matched Friedman test, with post-hoc Dunn’s multiple comparisons test, as indicated for each result. A *p* value of ≤ 0.05 was defined as statistically significant.

## 3 Results

### 3.1 Cytokine screening on mRNA and protein levels

**Figure 1:**
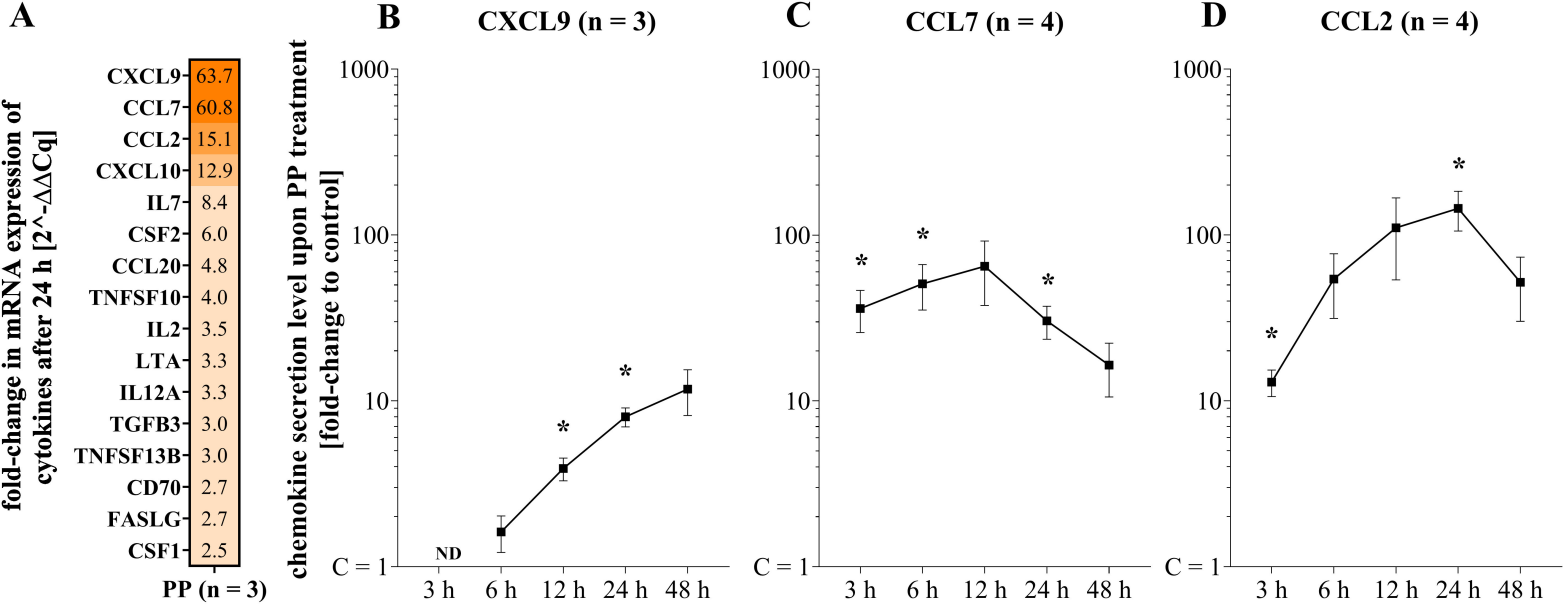
Cytokine expression and time dependent release by PBMCs after PP treatment **(A)** mRNA expression levels of cytokines with a fold-change higher than 2 compared to C (n = 3) measured by RT-qPCR; **(B-D)** CXCL9, CCL7 and CCL2 secretion levels after 3 h, 6 h, 12 h, 24 h and 48 h of PP incubation measured by ELISA; data is displayed as mean fold-change to C with SEM; data represents biological replicates of different donors; normally distributed by Shapiro-Wilk test; *p < 0.05 C *versus*. PP by 2-tailed, paired t-test per time point.

Innate and adaptive immune cells hypersecrete pro-inflammatory cytokines in the cytokine storm caused by SARS-CoV-2 infections (52). Using RTqPCR, we tested the RNA expression levels of 116 cytokines and compared them between PP-treated and untreated PBMCs. Fifty-one cytokines were expressed by all three donors in the control and the PP treatment. More than a two-fold increase in release was detected for 16 chemokines and cytokines compared to the control (C) in the PP-treated PMBCs (**Figure 1A**).

*CXCL9*, *CCL7*, and *CCL2* were the chemokines with the highest fold changes of mRNA expression levels after 24 h in response to PP treatment compared to C (**Figure 1A**). The protein release of these three chemokines was analyzed in the secretome of PBMCs after 3 h, 6 h, 12 h, 24 h, and 48 h of PP treatment via ELISA (**Figures 1B–D**). As the chemokine concentrations varied considerably between donors, data are displayed as fold changes relative to C per donor to facilitate interpretation of the changes due to PP treatment. Unless stated otherwise, the significance levels refer to the comparison with C.

PBMCs incubation with PP increased CXCL9 protein levels in the secretome to a 3.9 ± 0.6 (*p* < 0.05) fold change after 12 h and an 8.0 ± 1.1 (*p* < 0.05) fold change after 24 h. CCL7 protein secretion levels showed a 36.1 ± 10.3 (*p* < 0.05) fold increase after 3 h, a 50.9 ± 15.6 (*p* < 0.05) fold increase after 6 h, and a 30.4 ± 6.9 (*p* < 0.05) fold increase after 24 h of incubation with PP. CCL2 protein levels in the secretome of PP-treated PBMCs revealed a 13.0 ± 2.4 (*p* < 0.05) fold change after 3 h and a 144.7 ± 39.0 (*p* < 0.05) fold change after 24 h. Thus, as expected, PBMCs reacted to treatment with PP by increasing the mRNA expression and protein secretion of CXCL9, CCL7, and CCL2. The time to reach the peak mRNA expression levels varied between the cytokines, whereas an incubation period of 24 h resulted in maximum protein secretion of all three chemokines. With these functional alterations, CXCL9, CCL7, and CCL2 were identified as biomarkers for investigating the role of TAS2R signaling in PBMCs during the cytokine storm associated with SARS-CoV-2 infection.

### 3.2 PP-induced chemokine secretion after 24 h in PBMCs, T-cells (T), and T-cell-depleted PBMCs (w/oT)

**Figure 2:**
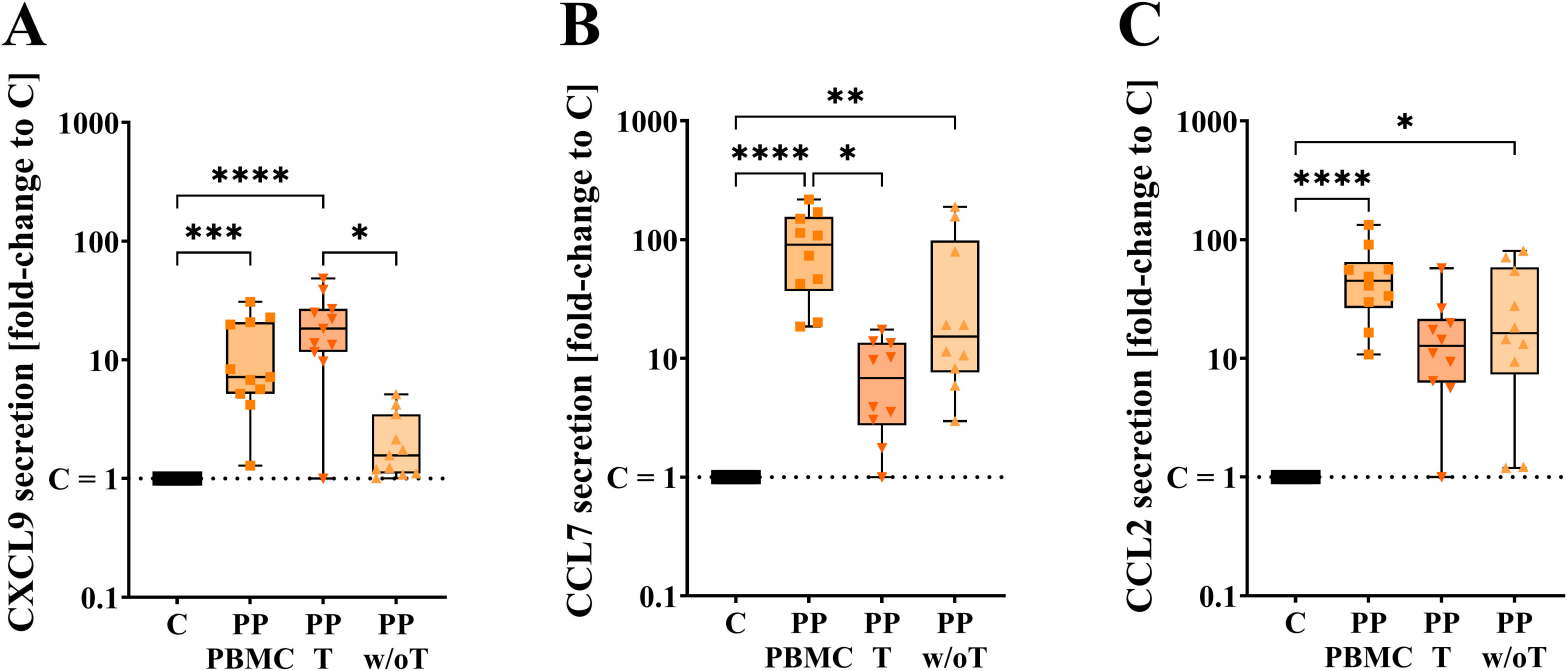
Chemokine secretion levels of **(A)** CXCL9 (n = 11), **(B)** CCL7 (n = 10), and **(C)** CCL2 (n = 10) of PMBCs, T-cells (T) and PBMCs depleted from CD3^+^ T-cells (w/oT) treated with PP for 24 h shown as fold-change to C per cell type; data is displayed as min to max box plot, all data points in the same treatment represent biological replicates of different donors; the same donors were used for comparison across different treatments; outlier test ROUT (Q = 1%); not all data normally distributed by Shapiro-Wilk test; non-parametric matched Friedman test, with post-hoc Dunn’s multiple comparisons test, *p < 0.05, **p < 0.01, ***p < 0.001, ****p < 0.0001.

PBMCs encompass around 61% T-cells, 13% B-cells, 15% NK cells, 9% monocytes, and 1% dendritic cells (23). T-cells are the most abundant cell type and play a significant role in adaptive immunity. To determine whether the chemokines are secreted primarily by T-cells, positive and negative T-cell isolation experiments were performed using the same cell concentration for PBMCs, T-cells (T), and PBMCs depleted of CD3^+^ T-cells (w/oT) from the same donors (**Figure 2**).

Immune cells are known to secrete cytotoxic mediators upon activation by PP (53). Thus, in line with previous reports, PP treatment decreased the cell viability [PBMC PP 85.3% ± 3.5% (*p* < 0.001 *versus* PBMC C), T PP 83.6% ± 4.4 (*p* < 0.001 *versus* T C), and w/oT PP 69.0% ± 1.3% (*p* < 0.001 *versus* w/oT C) viable; (**Supplementary Figure 1**)], with w/oT_-_cells impacted most by the isolation and treatment.

Twenty-four hours of treatment of PBMCs with PP increased the secretion of CXCL9, CCL7, and CCL2 to mean fold changes of 12.1 ± 2.9, 95.9 ± 21.4, and 51.7 ± 11.6 (*p* < 0.001 *versus* C), respectively (**Figure 2**). In T-cells of the same donors, 24 h incubation with PP induced CXCL9, CCL7, and CCL2 secretion levels to a 20.9 ± 4.1 (*p* < 0.0001 *versus* C) fold change, a 7.8 ± 1.9 (*p* > 0.05 *versus* C; *p* < 0.05 to PBMC) fold change, and a 16.9 ± 5.1 (*p* > 0.05 *versus* C) fold change, respectively. Therefore, while T-cells strongly contribute to the rise in CXCL9 levels, T-cells were not the main source for the increase in CCL2 and CCL7 secretion levels. PP treatment for 24 h of w/oT-cells resulted in increased CXCL9 secretion levels, shown by a 2.2 ± 0.4 (*p* > 0.05 *versus* C; *p* < 0.05 *versus* T) fold-change, CCL7 secretion levels, indicated by a 50.1 ± 21.6 (*p* < 0.01 *versus* C) fold change, and elevated CCL2 secretion levels by a 29.1 ± 9.1 (*p* < 0.05 *versus* C) fold rise. Consequently, w/oT-cells mainly help secrete CCL2 and CCL7, with a lesser role in secreting CXCL9.

Overall, the secretion levels of CXCL9, CCL7, and CCL2 protein upon treatment with PP were increased in PBMCs, but not consistently in T or w/oT-cells. Thus, the following experiments were continued in PBMCs comprising different cell types.

### 3.3 Co-incubation with TAS2R interaction partner HED suppresses chemokine secretion after 24 h

**Figure 3:**
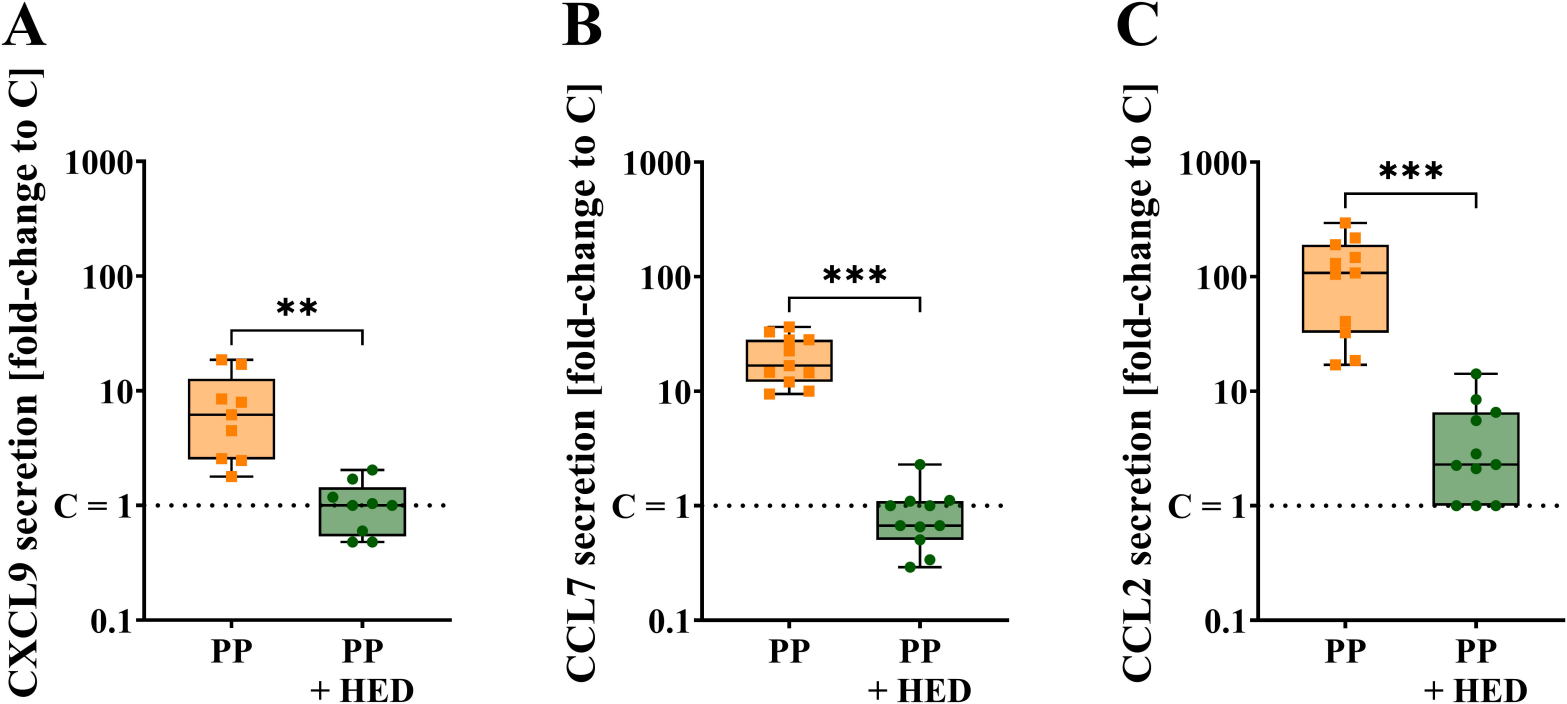
Chemokine secretion levels for **(A)** CXCL9 (n = 9), **(B)** CCL7 (n = 11), and **(C)** CCL2 (n = 11) in PBMCs after 24 h of incubation with and without PP alone or PP and HED; data is displayed as min to max box plot, mean values are given above plot, all data points in the same treatment represent biological replicates of different donors; the same donors were used for comparison across different treatments; outlier test ROUT (Q = 1%); not all data normally distributed by Shapiro-Wilk test; non-parametric matched Friedman test, with post-hoc Dunn’s multiple comparisons test. **p < 0.01, ***p < 0.001.

To assess whether TAS2Rs are potentially involved in the chemokine response in PBMCs upon PP treatment, PBMCs were incubated with PP with and without the broadly tuned TAS2R interaction partner HED for 24 h (**Figure 3**). Notably, HED is an agonist to TAS2R14 and -39 (46), and an antagonist to TAS2R31, -43, -50, and -20 (45).

After 24 h of incubation, PP-treated PBMCs (78.1% ± 4.1%) and cells co-incubated with PP+HED (76.9% ± 4.2%) did not differ in viability compared to untreated control PBMCs (**Supplementary Figure 2**). Therefore, the results obtained are derived from the various treatments administered and do not stem from a discrepancy in survival rates.

Upon PP treatment for 24 h, CXCL9, CCL7, and CCL2 secretion levels showed a mean 7.7 ± 2.1, 20.5 ± 2.9, and 118.4 ± 27.2 fold increase per donor, respectively. In contrast, when PBMCs were co-incubated with HED and PP for 24 h, the chemokine release was changed by a 1.1 ± 0.2 fold for CXCL9 (**Figure 3A**), a 0.9 ± 0.2 fold for CCL7 (**Figure 3B**), and a 4.3 ± 1.2 fold for CCL2 (**Figure 3C**). When percent changes were calculated, the PP-induced CXCL-9, CCL-7, and CCL-2 chemokine secretions decreased by a mean of 80.2% ± 3.7% (*p*<0.01), 95.7% ± 0.4% (*p*<0.001), and 95.0% ± 1.1% (*p*<0.001), respectively, by co-incubation of PBMCs with PP and HED. These findings suggest a functional involvement of TAS2Rs in the PP-induced chemokine responses observed in PBMCs.

### 3.4 PP effect on mRNA expression levels of TAS2Rs on PBMCs

To confirm the functional involvement of TAS2Rs in cytokine secretion by PBMCs, we investigated whether specific *TAS2R* mRNA expression levels are altered after PP treatment to identify potential targets for further investigation by siRNA knock-down experiments.

**Figure 4:**
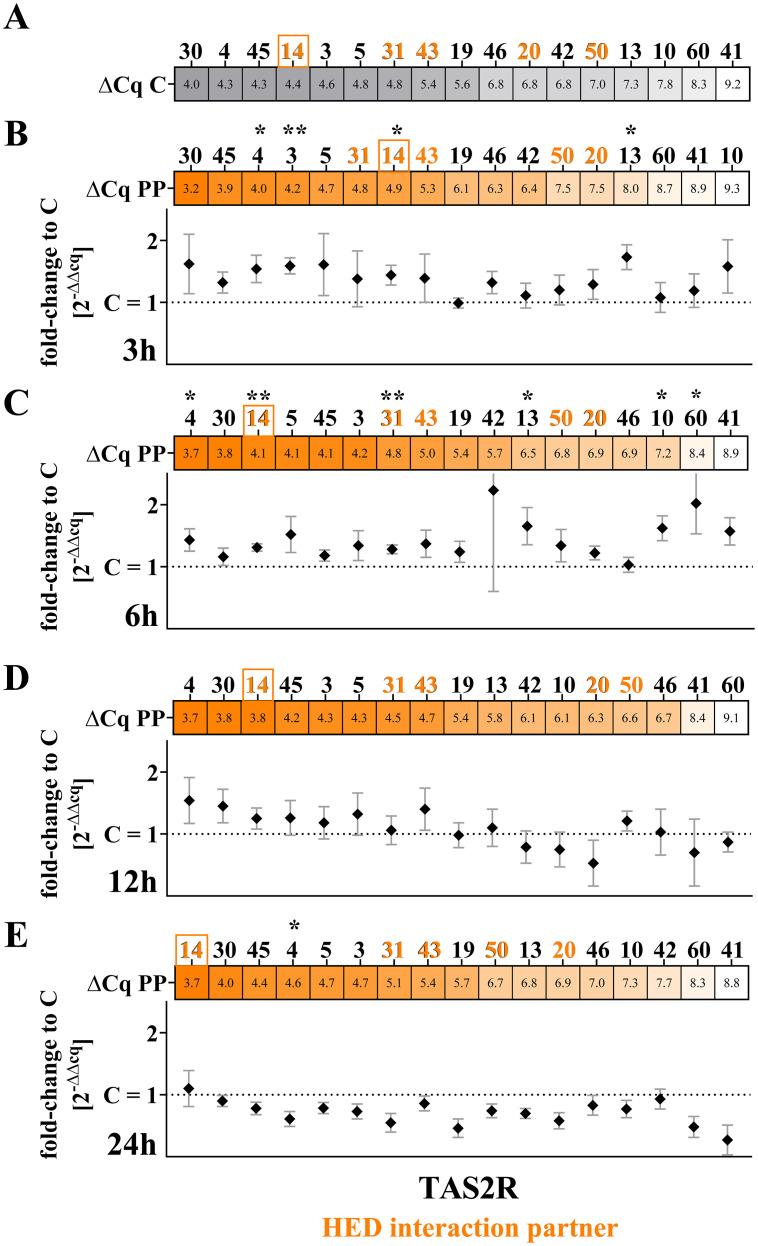
mRNA expression levels of bitter taste receptors in PBMCs (n ≥ 3); Δcq values show the relative abundance of the TAS2R mRNA transcripts ordered from highest relative abundance to lowest (left to right). Known HED interaction partners are highlighted in orange. TAS2R14 mRNA is highly abundant in PBMCs (orange square). **(A)** Heat map of the average Δcq of TAS2Rs in C PBMCs; **(B-E)** PP-induced changes in TAS2R mRNA expression in PBMCs treated for **(B)** 3 h, **(C)** 6 h, **(D)** 12 h and **(E)** 24 h; 2^-ΔΔct^ values depict the fold-change to C after PP treatment per time point; normally distributed Shapiro-Wilk test, *p < 0.05, **p < 0.01, C *versus*. PP by 2-tailed, paired t-test.

First, mRNA expression was quantified by qRT-PCR after 3 h, 6 h, 12 h, and 24 h of PP incubation. Of the 25 tested *TAS2Rs*, 17 were stably expressed (ct < 37) in at least 3 donors at all time points and treatments (**Figure 4**). Due to their low expression (ct > 37) in fewer than 3 donors, results for TAS2R1, -7, -8, -9, -16, -38, -39, and -40 are not shown. The four receptors with the highest relative mRNA abundance in untreated C were, in order, *TAS2R30*, *TAS2R4*, *TAS2R45*, and *TAS2R14* (**Figure 4A**).

PP induced transient, time-dependent changes in TAS2R mRNA expression. After 3 h of incubation, expression levels of TAS2R4, TAS2R3, TAS2R14, and TAS2R13 were increased, reaching 1.5 ± 0.2-fold (*p* < 0.05), 1.6 ± 0.1-fold (*p* < 0.01), 1.4 ± 0.2-fold (*p* < 0.05) and 1.7 ± 0.2-fold (*p* < 0.05), respectively (**Figure 4B**). At 6 h, PP treatment resulted in a broader induction pattern, with upregulation of TAS2R4, TAS2R14, TAS2R31, TAS2R13, TAS2R10, and TAS2R60, showing fold increases of 1.4 ± 0.2 (*p* < 0.05), 1.3 ± 0.1 (*p* < 0.01), 1.3 ± 0.1 (*p* < 0.01), 1.7 ± 0.3 (*p* < 0.05), 1.6 ± 0.2 (*p* < 0.05), and 2.0 ± 0.5 (*p* < 0.05), respectively (**Figure 4C**). In contrast, 12 h of PP exposure did not affect TAS2R transcript levels (**Figure 4D**). After 24 h, TAS2R4 expression was reduced to 0.6 ± 0.1-fold relative to control (*p* < 0.05) (**Figure 4E**).

This indicates that treatment of PMBCs with SARS-CoV-2 peptides alters the expression levels of *TAS2Rs*, suggesting that these receptors are involved in regulating cytokine expression. In particular, the results for *TAS2R14* mRNA expression upon PP treatment were noteworthy since its mRNA expression levels were raised after 3 h and 6 h of PP-treatment, as well as its relative abundance increased over PP incubation time until it was the *TAS2R* with the highest relative abundance after 24 h, with a Δcq value of 3.6 ± 0.3. Hence, we performed siRNA *TAS2R14* knock-down (*TAS2R14kd*) experiments in the next step.

### 3.5 Knock-down of TAS2R14 reveals its involvement in chemokine secretion in PBMCs

To investigate the functional role of TAS2R14 in the PP-induced chemokine secretion, an siRNA knock-down (kd) of *TAS2R14* was performed. Mean efficiency was 42.2% ± 4.5% (n = 11) for the *TAS2R14kd* PP-treated cells compared to mock C measured by RT-qPCR.

Viability was 84.8 ± 4.3% in GAPDHkd C and 71.7 ± 1.2% in mock C PBMCs (**Supplementary Figure 3**). PP treatment reduced viability to 48.7 ± 1.7% in mock-transfected cells (*p* < 0.0001 *versus* GAPDHkd C; *p* < 0.001 *versus* mock C) and to 54.6 ± 2.0% in TAS2R14kd PBMCs (*p* < 0.05 *versus*. GAPDHkd C). Viability did not differ between PP-treated mock and TAS2R14kd PBMCs (*p* > 0.05), indicating that the observed effects are independent of differential cell survival.

**Figure 5:**
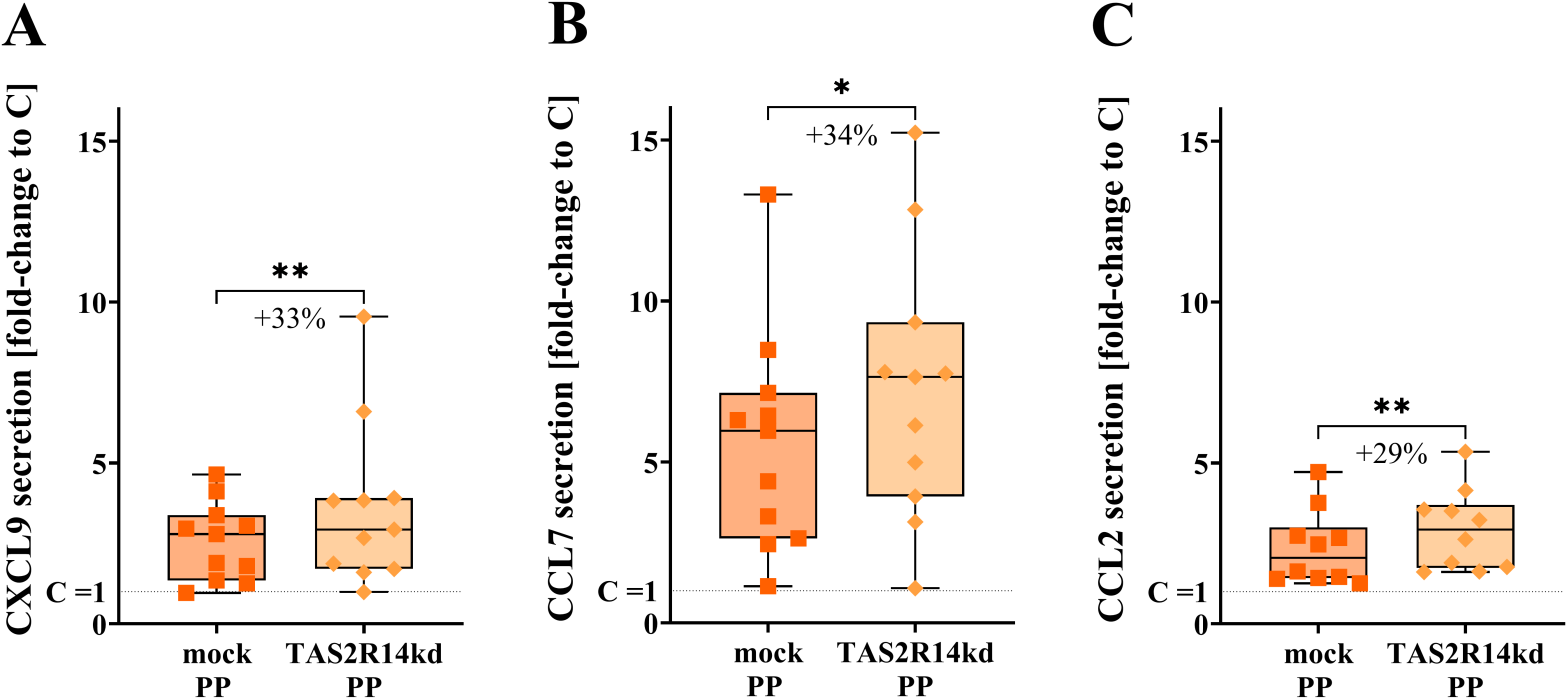
Chemokine secretion levels of **(A)** CXCL9 (n = 11), **(B)** CCL7 (n = 11), and **(C)** CCL2 (n = 10) of mock and TAS2R14kd PMBCs treated with PP for 24 h shown as fold-change to mock C; data is displayed as min to max box plot, mean values are given above plot, all data points in the same treatment represent biological replicates of different donors; the same donors were used for comparison across different treatments; outlier test ROUT (Q = 1%); not all data normally distributed by Shapiro-Wilk test; Wilcoxon matched-pairs signed rank test, *p < 0.05, **p < 0.01.

In mock-transfected 24 h PP- treated PBMCs, CXCL9 secretion upon PP treatment increased by a 2.6 ± 0.4 fold change compared to mock C (**Figure 5A**), which increased to a mean of 3.6 ± 0.8 (*p* < 0.01) fold change *versus* mock C in PBMCs with *TAS2R14kd* after 24 h of PP incubation, amounting to a rise of 32.5 ± 11.3%.

CCL7 secretion levels were raised to a 5.6 ± 1.0 mean fold change *versus* mock C for mock-transfected PBMCs upon 24 h of PP treatment, which was increased to a 7.3 ± 1.3 (*p* < 0.05) fold change compared to mock C when *TAS2R14* was knocked down in the same donors (**Figure 5B**), equivalent to an increase of 34.1 ± 12.0%.

CCL2 secretion levels upon 24 h of PP treatment increased 2.4 ± 0.4-fold compared to mock C after mock-transfection, while *TAS2R14kd* led to a 2.9 ± 0.4 (*p* < 0.01) fold increase compared to mock C (**Figure 5C**), which is equivalent to a rise of 29.3 ± 8.8%.

This rise of PP-induced release of chemokines in *TAS2R14kd* PBMCs demonstrates a functional role of TAS2R14 in the chemokine immune response of primary blood immune cells. While the role of other receptors cannot be excluded, we suggest TAS2R14 as a promising receptor for immune system regulation in SARS-CoV-2 infections.

## 4 Discussion

We hypothesized that TAS2Rs represent novel immunomodulatory therapeutic targets in the proinflammatory cytokine immune response induced by SARS-CoV-2 peptides in human PBMCs. This hypothesis builds upon previous findings on the hyperproduction of cytokines (4–7) and taste loss (25) in COVID-19 patients, the bitterness of anti-viral drugs (33–37), the protein-protein interaction of SARS-CoV-2 with TAS2Rs (50), and the potential of TAS2Rs expressed on blood immune cells (54) as modulators of cytokine secretion (55–57). Our results demonstrate a reduction of the SARS-CoV-2 PP-induced CXCL9, CCL7, and CCL2 burst of PBMCs by HED, which is an antagonist to TAS2R31, -43, -50, and -20 (45) and an agonist of TAS2R14 and -39 (46). The functional role of TAS2R14 in this response was confirmed by a siRNA *TAS2R14* knock-down of PBMCs, leading to increased cytokine secretion upon PP treatment.

As previously demonstrated (5–7), we have shown that treatment of PBMCs with PP results in a marked increase in CXCL9, CCL7, and CCL2 cytokine release. This result closely resembles the hypersecretion of cytokines of patients with severe SARS-CoV-2 infection (5–7), although it does not preclude the involvement of additional cytokines. However, in line with our hypothesis, the cytokine burst observed during SARS-CoV-2 infection, which might contribute to the loss of bitter taste (26, 58), together with the fact that several antiviral drugs have been shown to interact with TAS2Rs (33–37), suggests that TAS2Rs may represent promising therapeutic targets for the development of novel anti-inflammatory interventions. This rationale is substantiated by the ensuing findings, which demonstrate that *TAS2R14* mRNA levels of PBMCs were *(i)* increased after PP treatment for 3 h and 6 h, *(ii)* represented the most abundant *TAS2R* mRNA after 24 h PP exposure, and (iii) determined functional changes in CXCL9, CCL7 and CCL2 secretion, as demonstrated by a *TAS2R14*-specific siRNA-knock down approach. In this context, the 29% - 34% increase of chemokine secretion upon *TAS2R14kd* was compelling, given the siRNA knock-down efficacy of 42%, which falls within the range of other siRNA nucleofector-based approaches reported in primary blood immune cells (59). While we cannot exclude the role of other receptors and pathways, TAS2R14 clearly demonstrated its involvement in regulating a hyperinflammatory state in PBMCs. Therefore, TAS2R14 is especially promising as a target for reducing an excessive inflammatory cytokine response during SARS-CoV-2 infection.

In reviewing the literature, no other data were found on the association between TAS2Rs and cytokine modulation in viral infections. However, our results are supported by the activation of TAS2R14 by the bitter-tasting anti-viral drug ritonavir, which was used in combination with nirmatrelvir in SARS-CoV-2 infections (35–37). Recently, two binding sites of ritonavir were suggested in TAS2R14, which was validated by calcium mobilization using mutated versions of this receptor in a HEK transfection model (37). These results provide further support for the hypothesis that TAS2R14 is a therapeutic target in viral infections. Importantly, future work is needed to evaluate the impact of TAS2R14 on anti-viral drug treatment, as bitter taste and TAS2R activation do not prove anti-viral effects.

Unlike the lack of studies on the role of TAS2Rs in viral infections, there is evidence regarding the anti-inflammatory properties of TAS2R14 in bacterial infections, induced by, for example, LPS. Notably, TAS2R14 is the most broadly tuned TAS2R, interacting with a wide variety of different compounds (41, 60, 61). In human PBMCs, the quorum-sensing molecule 3−oxo−C12:2 homoserinelactone reduced the LPS-induced pro-inflammatory TNF-α secretion dose-dependently (25 - 100 µM) and activated six TAS2Rs, among them TAS2R14, in a calcium mobilization assay performed in HEK293T reporter-cells (62). Also, the TAS2R14 agonist carisoprodol reduced LPS (10 ng/mL) -induced IL-10, TNF-α, CCL3, and CXCL8 secretion in human lung macrophages of cancer patients *ex-vivo* at a concentration of 0.3 mM and 1 mM, respectively (32), which is another example of the anti-inflammatory capabilities of TAS2R14 on human immune cells.

The anti-inflammatory properties of the TAS2R14 agonist HED have been previously reported in LPS-treated macrophage RAW264.7 cells, and are proposed to be involved in the decrease of IL-6 and TNF-α through the nuclear factor-kappa B (NF-κB) signaling pathway (63). These findings align with our results in the viral SARS-CoV 2 context, which showed that co-incubation of PBMCs with HED and PP suppressed the PP-induced burst of CXCL9, CCL7, and CCL2.

Flufenamic acid, a well-established nonsteroidal anti-inflammatory drug (64), is, like HED, a potent agonist of TAS2R14 (41) of which the cryo–electron microscopy structure has been published recently (65). Moreover, decreased mRNA expression levels of bacterial LPS-induced IL-1β, IL-2, IL-6, IP-10, CCL2, and TNF-α were reported in airway smooth muscle cells upon treatment with flufenamic acid (66). These anti-inflammatory effects of flufenamic acid might accord with our results, as TAS2R14 was previously reported to be the only TAS2R that flufenamic acid activates (41). However, since flufenamic acid influences numerous physiological processes through a broad range of targets (67–69), further investigations are needed to determine whether its anti-inflammatory effects are at least partly mediated via TAS2R14 in bacterial and viral infections.

Finally, it is important to consider the following possible biases in the presented study. Instead of infecting cells with intact viruses, we used an established model to stimulate immune cells in these *ex-vivo* experiments (48, 49) by applying SARS-CoV-2 specific 15-mer peptide pools overlapping by 11 amino acids. Also, the buffy coats from healthy anonymous adults used in this study lacked information on demographics or history of SARS-CoV-2. Thus, the unknown antibody status and representativeness of our results for the population are limitations of this study. High inter-individual differences, increasing population immunity since the start of the pandemic, with several possible reinfections from different virus variants, varying immunologic states at the time of blood donation, and small sample sizes somewhat limit the power of these results. To adjust for interindividual donor differences and the known chemokine-stimulating effect of nucleofection (70), individual values were normalized relative to the control values per donor, allowing us to focus on the differences due to the treatment and/or TAS2R14 knockdown. That said, another limitation of this study might be that the contribution of individual cell types was not investigated. On the other hand, our approach considers the bidirectional interplay between innate and adaptive immune responses (71). A previous study compared the response of T-cells to PP treatment between COVID-19 patients with different disease severities and unexposed donors and found no differences in magnitude or functionality of the T–cell response before and after recovery, as well as between deceased and recovered critical COVID-19 patients (49). Thus, T–cells are unlikely to be solely responsible for the differences in COVID-19 disease severity, supporting the use of PBMCs instead of T–cells alone.

In conclusion, the present study demonstrates that the production of pro-inflammatory chemokines, such as CXCL9, CCL7, and CCL2, from primary immune cells in response to SARS-CoV-2 peptides can be reduced by the anti-inflammatory compounds targeting TAS2R14. The presented anti-inflammatory effects of the TAS2R14 agonist HED were confirmed by TAS2R14-specific siRNA knock-down experiments, demonstrating a receptor-dependent activity. Overall, these findings highlight a promising therapeutic potential of TAS2R14 agonists for mitigating inflammatory complications associated with viral SARS-CoV-2 infections.

## Data Availability

The raw data supporting the conclusions of this article will be made available by the authors, without undue reservation.
