# Supplementary material for: Homoeriodictyol, targeting the bitter taste receptor TAS2R14, lowers the secretion of pro-inflammatory chemokines upon treatment with SARS-CoV-2 peptide pools in human peripheral blood mononuclear cells
Source: Front Immunol. 2026 Feb 3;17:1771794. doi: 10.3389/fimmu.2026.1771794 (PMC12909574; doi:10.3389/fimmu.2026.1771794)
Supplement: Supplementary file 1 [file DataSheet1.pdf]

Supplementary Material

1 Supplementary Figures

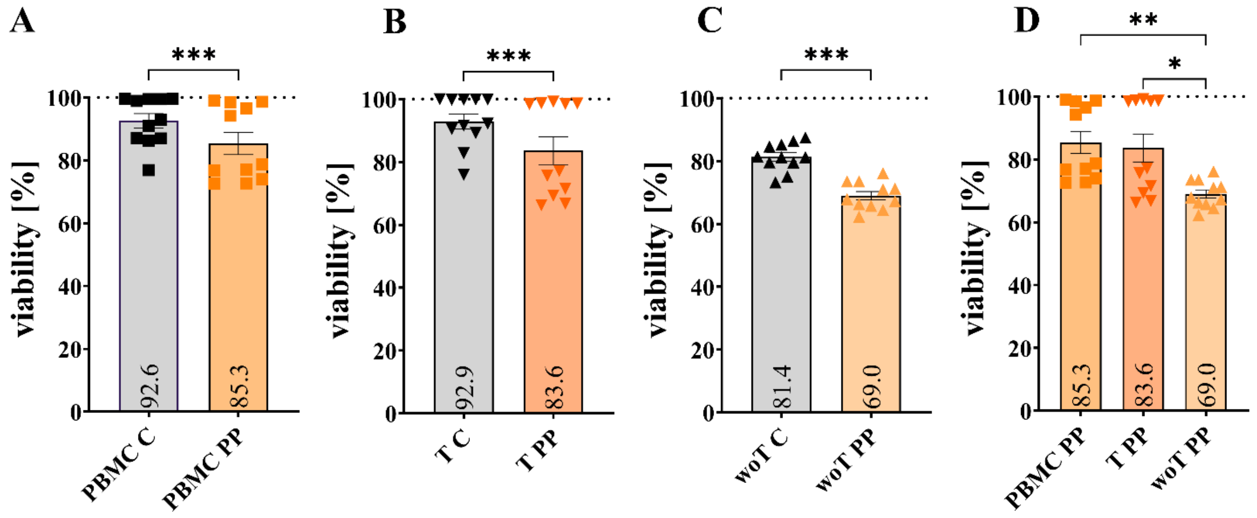

Supplementary Figure 1. Viability of (**A**) PBMCs, (**B**) T-cells and (**C**) PBMCs depleted from $CD3^+$ T-cells (woT) without (C) and with PP-treatment for 24 h and (**D**) comparison of viability of PP treated cells; determined by flow cytometry with propidium iodide staining (n = 11); data is displayed as scatter plot with bar of mean with SEM; outlier test ROUT (Q = 1 %); not all data normally distributed by Shapiro-Wilk test; (**A-C**) Wilcoxon matched-pairs signed rank test, (**D**) Friedman test, Dunn's multiple comparisons test; * $p < 0.05$, ** $p < 0.01$, *** $p < 0.001$.

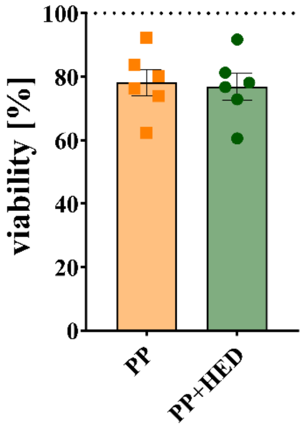

Supplementary Figure 2. Viability of PBMCs treated with PP alone or in combination with HED for 24 h; determined by flow cytometry with propidium iodide staining (n = 6); data is displayed as scatter plot with bar of mean with SEM; outlier test ROUT (Q = 1 %); data normally distributed by Shapiro-Wilk test; paired t-test (*p* > 0.05).

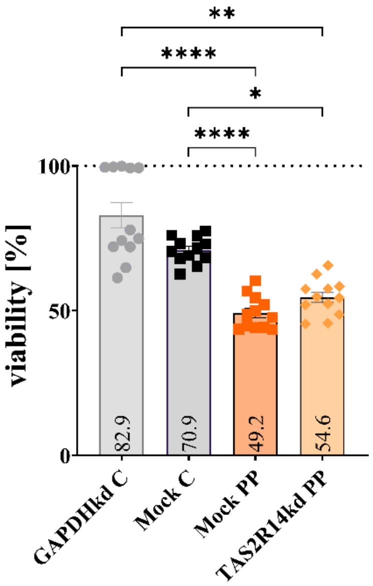

Supplementary Figure 3. Viability of siRNA nucleofected PBMCs without (C) and with PP-treatment for 24 h; determined by flow cytometry with propidium iodide staining (n = 11); data is displayed as scatter plot with bar of mean with SEM; outlier test ROUT (Q = 1 %); not all data normally distributed by Shapiro-Wilk test; Friedman test, Dunn's multiple comparisons test; * p < 0.05, *** p < 0.001, **** p < 0.0001.
